# Supplementary material for: Gut Microbiome of a Multiethnic Community Possessed No Predominant Microbiota
Source: Microorganisms. 2021 Mar 29;9(4):702. doi: 10.3390/microorganisms9040702 (PMC8065435; doi:10.3390/microorganisms9040702)
Supplement: Supplementary file 1 [file microorganisms-09-00702-s001.zip › microorganisms-1123010-supplementary.docx]

Gut Microbiome of a Multiethnic Community Possessed No Predominant Microbiota

Wei Wei Thwe Khine ^1,2^, Anna Hui Ting Teo ^1^, Lucas Wee Wei Loong ^3^, Jarett Jun Hao Tan ^3^,
Clarabelle Geok Hui Ang ^3^, Winnie Ng ^3^, Chuen Neng Lee ^4^, Congju Zhu ^3^, Quek Choon Lau ^3^ and
Yuan Kun Lee ^1,4,^*

**Supplemental Materials**

**Supplementary Figures Legends**


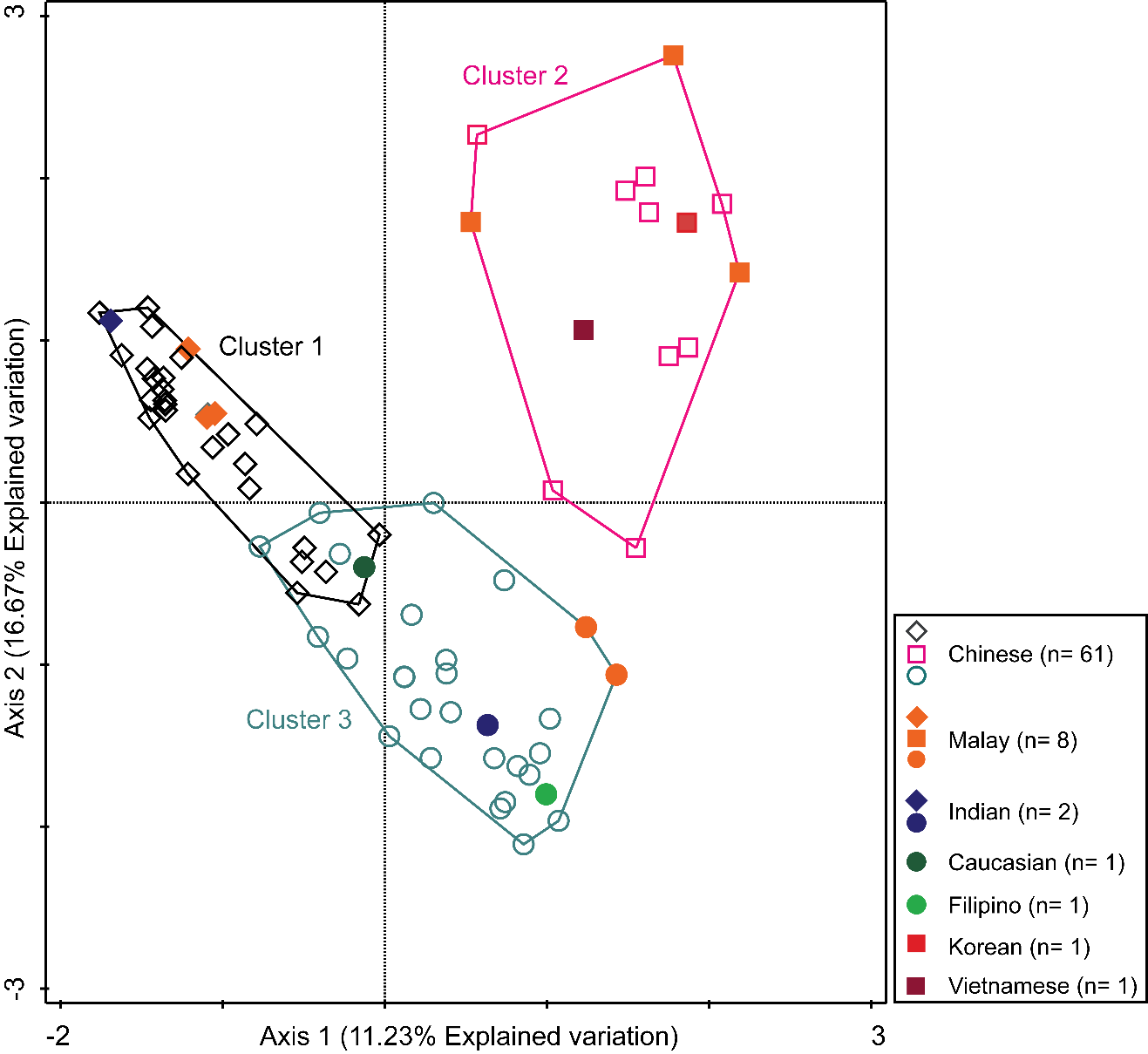


Figure S1. Composition of faecal microbiome in different ethnicities of three clusters at baseline. Species biplots of distance-based redundancy analysis (db-RDA) based on the square root Bray-Curtis distance matrix showed the distribution of faecal bacterial genera. The percentage of axes explain the compositional variation of the respective axis. The distances between each pair of clusters were tested for the significant difference by permutational multivariate analysis of variance (PERMANOVA) and multiple comparison pairwise test of Bonferroni at 4999 permutations. Different symbols and colours represent the types of clusters and ethnicities. Numbers of samples in parenthesis.


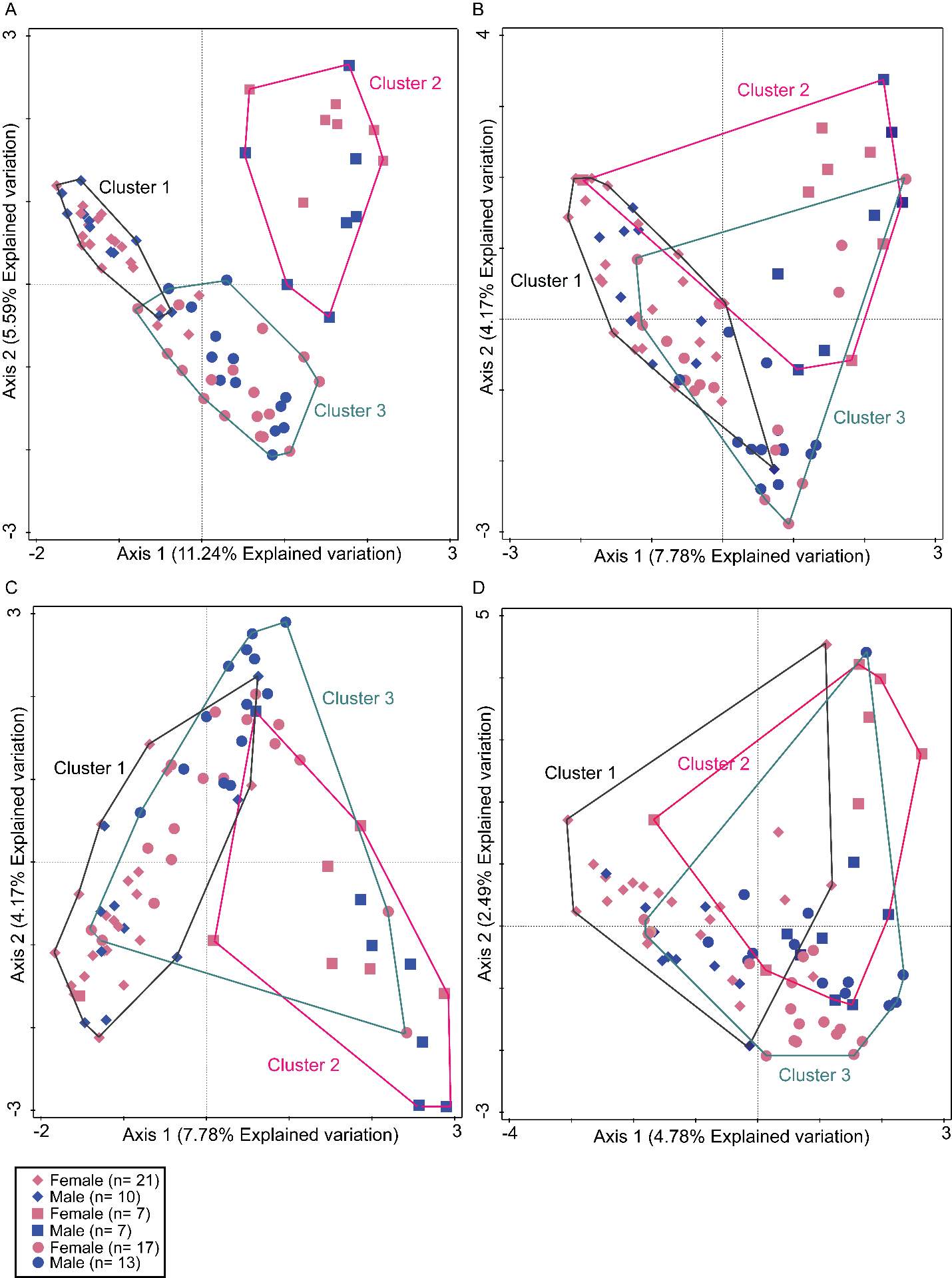


Figure S2: Composition of faecal microbiome in both genders in three clusters at (A) baseline, (B) Timepoint 2, (C) Timepoint 3, and (D) Timepoint 4. Species biplots of distance-based redundancy analysis (db-RDA) based on the square root Bray-Curtis distance matrix showed the distribution of faecal bacterial genera. The percentage of axes explain the compositional variation of the respective axis.The distances between each pair of clusters were tested for the significant difference by permutational multivariate analysis of variance (PERMANOVA) and multiple comparison pairwise test of Bonferroni at 4999 permutations. Different symbols and colours represent the types of clusters and genders. Numbers of samples in parenthesis.


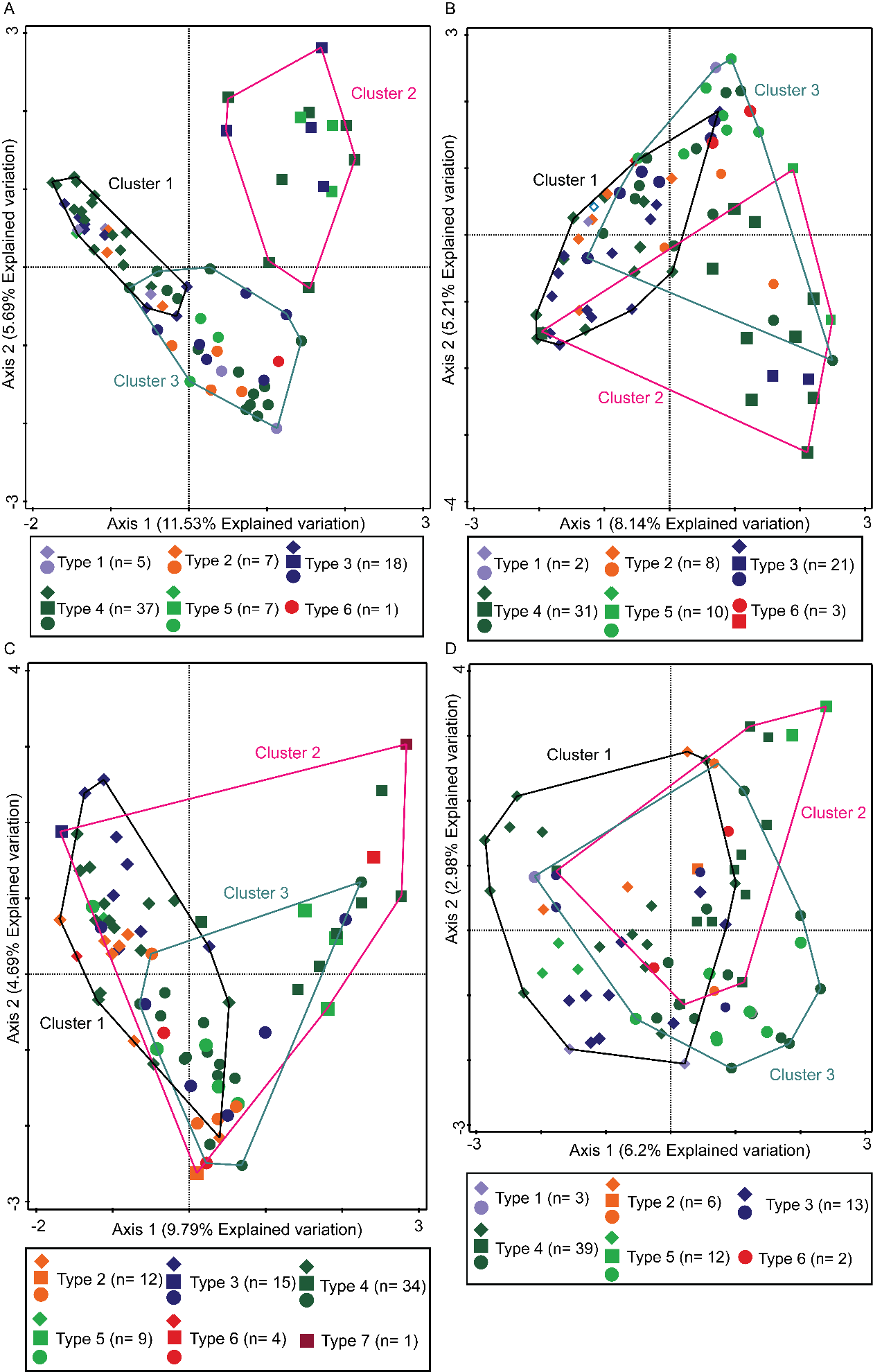


Figure S3: Composition of faecal microbiome in different Bristol stool scale types in three clusters at (A) baseline, (B) Timepoint 2, (C) Timepoint 3, and (D) Timepoint 4. Species biplots of distance-based redundancy analysis (db-RDA) based on the square root Bray-Curtis distance matrix showed the distribution of faecal bacterial genera. The percentage of axes explain the compositional variation of the respective axis. The distances between each pair of clusters were tested for the significant difference by permutational multivariate analysis of variance (PERMANOVA) and multiple comparison pairwise test of Bonferroni at 4999 permutations. Different symbols and colours represent the types of clusters and Bristol stool scale types. Numbers of samples in parenthesis.


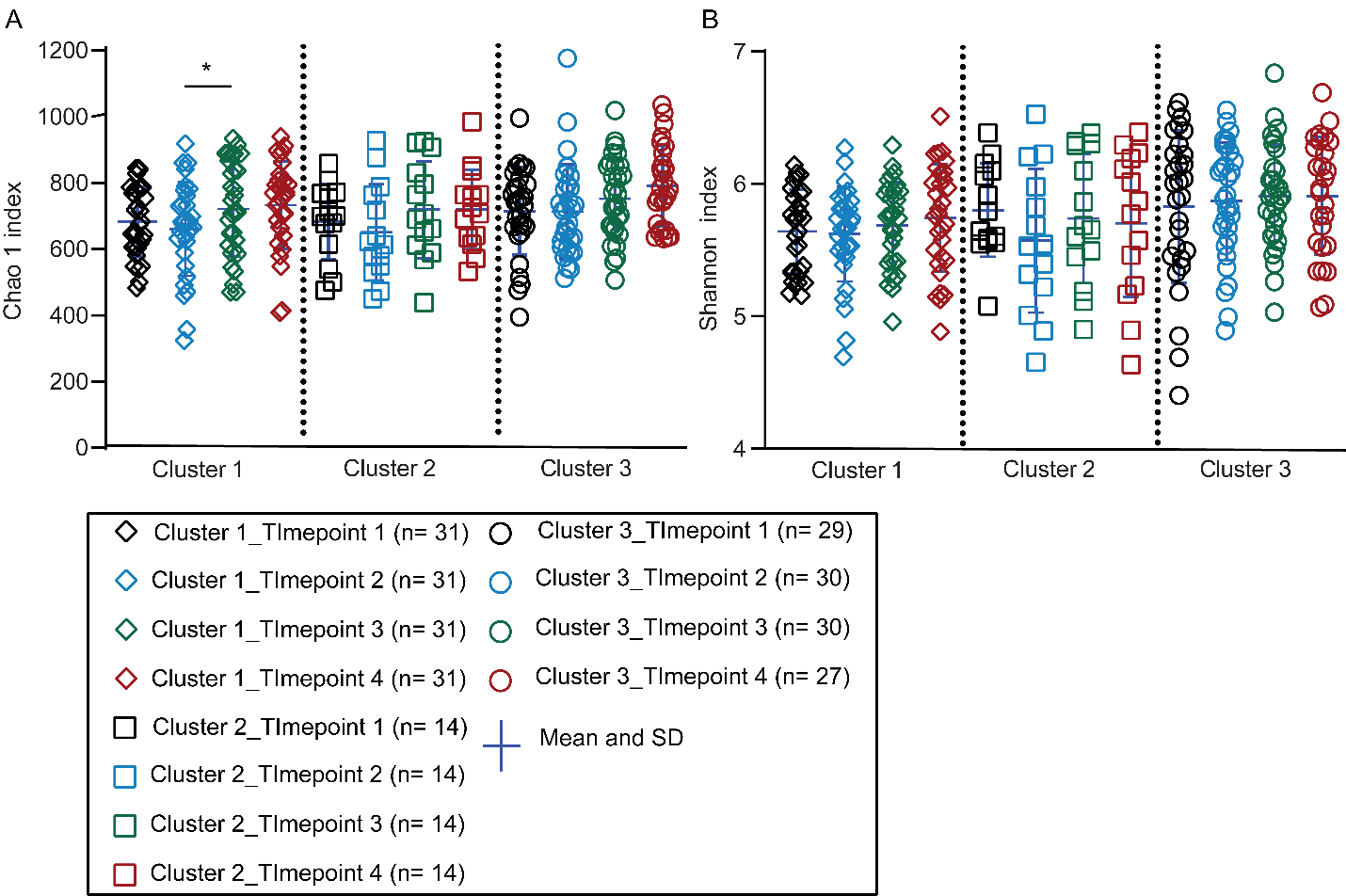


Figure S4: (A) Chao 1’s and (B) Shannon’s indices of alpha diversity comparing four time points of three clusters. Different symbols and colours represent different clusters and time points. Means and SD of indices are presented. One-way analysis of variance (ANOVA) followed by Bonferroni multiple comparisons tests were applied and the indices which were significantly different between the clusters at baseline or between the time points of each cluster were presented as *p ≤0.05. Timepoint 1= baseline; 14 days after washout, Timepoint 2= first 7 days after ingestion, Timepoint 3= second 7 days after ingestion, Timepoint 4= follow-up; 14 days after non-ingestion, SD= standard deviation. Numbers of samples in parenthesis.


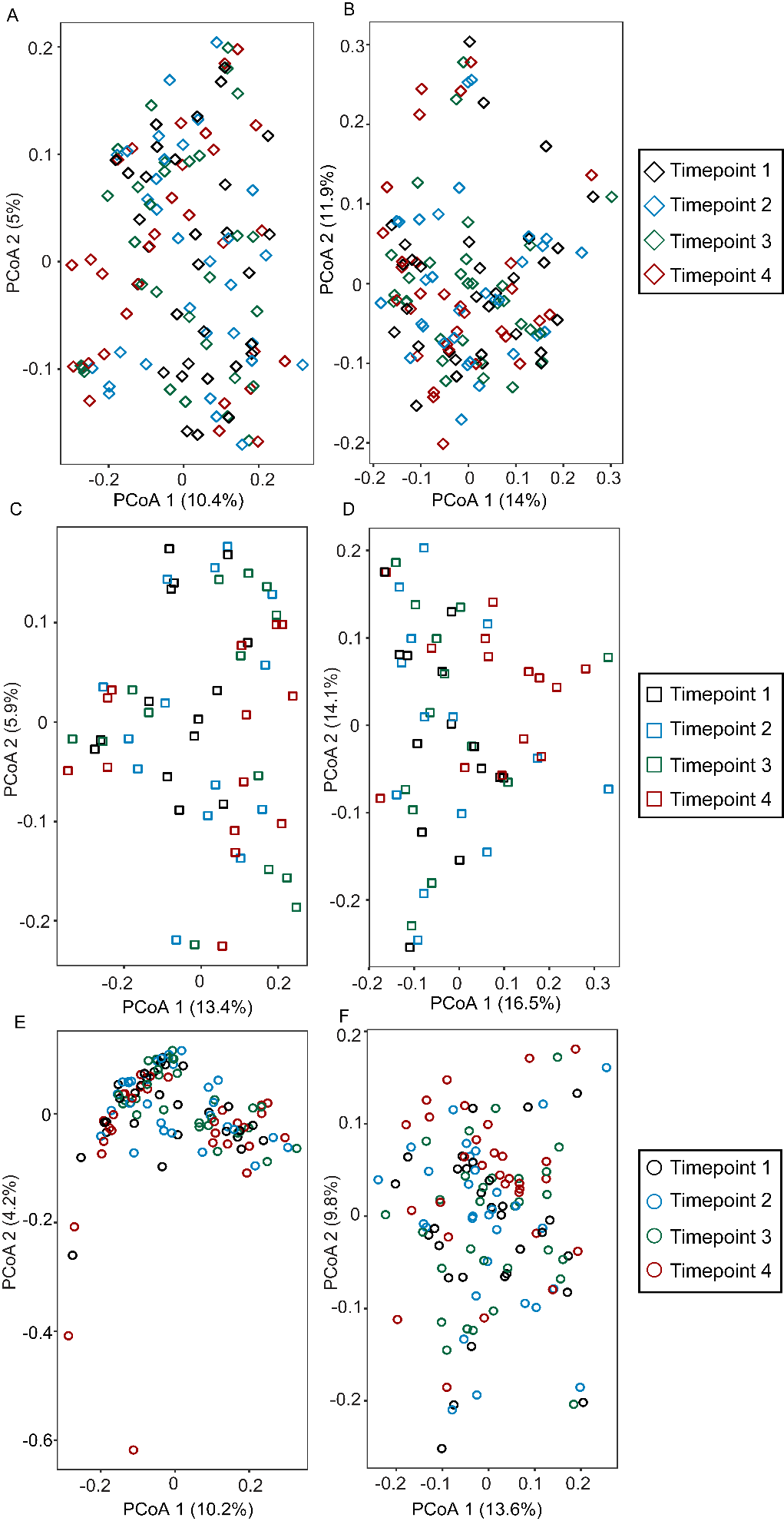


Figure S5: (A, C and E) Unweighted and (B, D and F) weighted Unifrac principal coordinates analysis (PCoA) of beta diversity comparing three clusters for (A and B) Timepoint 2, (C and D) Timepoint 3 and (E and F) Timepoint 4. Different symbols and colours represent different clusters. The distances between each cluster were tested for the significant difference by permutational multivariate analysis of variance (PERMANOVA) and multiple comparison pairwise test of Bonferroni at 4999 permutations. Timepoint 2= first 7 days after ingestion, Timepoint 3= second 7 days after ingestion, Timepoint 4= follow-up; 14 days after non-ingestion.


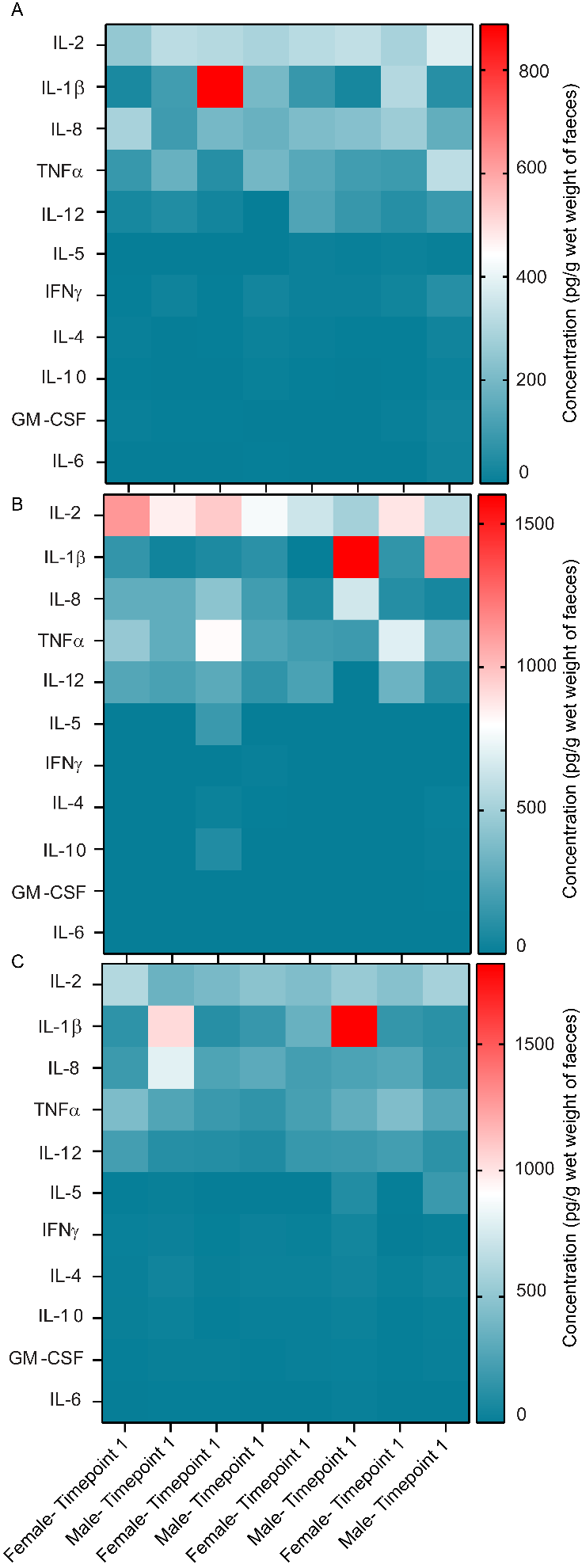


Figure S6: Comparison of concentration of faecal water cytokines (pg/g of wet weight of faeces) of (A) Cluster 1 (B) Cluster 2 (C) Cluster 3 across the time points. Means of concentration are presented. No significant difference in the cytokine levels between the time points were found by mixed-effects model followed by Bonferroni multiple comparisons tests. IL= Interleukin, TNF= Tumour necrosis factor, IFN= Interferon, GM-CSF= Granulocyte-macrophage-stimulating factor. Timepoint 1= baseline; 14 days after washout, Timepoint 2= first 7 days after ingestion, Timepoint 3= second 7 days after ingestion, Timepoint 4= follow-up; 14 days after non-ingestion.

**Supplementary Tables**

Table S1: Results of permutational multivariate analysis of variance (PERMANOVA) test comparing different clusters of major faecal microbiota at baseline

| Pairs | Sums of squared | F.Model | R^2^ | p-value | p-adjusted |
| --- | --- | --- | --- | --- | --- |
| Cluster 1 vs Cluster 2 | 0.513 | 10.048 | 0.189 | ***0.0002 | ***0.0006 |
| Cluster 1 vs Cluster 3 | 0.435 | 7.465 | 0.112 | ***0.0002 | ***0.0006 |
| Cluster 2 vs Cluster 3 | 0.341 | 5.749 | 0.120 | ***0.0002 | ***0.0006 |

R^2^ and p values shown were calculated by Adonis test and 4999 permutations using the square root of Bray-Curtis distance of the relative abundances of bacterial genera. p values were adjusted by Bonferroni’s multiple pairwise tests and presented as *** p < 0.001.

Table S2: Relative abundance of OTUs of major (>1% of total OTUs) faecal bacterial genera at baseline

| Bacteria | Cluster 1 | Cluster 2 | Cluster 3 |
| --- | --- | --- | --- |
| *Bacteroides* | 0.305 | 0.082 | 0.117 |
| *Blautia* | 0.116 | 0.116 | 0.159 |
| *Lachnospiraceae* | 0.142 | 0.110 | 0.116 |
| *Faecalibacterium* | 0.094 | 0.101 | 0.122 |
| *Ruminococcaceae* | 0.033 | 0.061 | 0.084 |
| *Prevotella* | 0.002 | 0.213 | 0.011 |
| *Coprococcus* | 0.029 | 0.041 | 0.059 |
| *Bifidobacterium* | 0.035 | 0.031 | 0.049 |
| *[Ruminococcus]* | 0.029 | 0.013 | 0.027 |
| *Ruminococcus* | 0.008 | 0.008 | 0.041 |
| *Dorea* | 0.019 | 0.010 | 0.013 |
| *Collinsella* | 0.009 | 0.017 | 0.016 |
| Clostridiales | 0.009 | 0.012 | 0.017 |
| *Streptococcus* | 0.011 | 0.012 | 0.013 |
| *Lachnospira* | 0.016 | 0.007 | 0.010 |
| *Megamonas* | 0.015 | 0.019 | 0.004 |
| *[Eubacterium]* | 0.002 | 0.010 | 0.020 |
| *Megasphaera* | 0.014 | 0.008 | 0.009 |
| *Oscillospira* | 0.008 | 0.007 | 0.009 |
| *Enterobacteriaceae* | 0.006 | 0.014 | 0.004 |
| *Parabacteroides* | 0.009 | 0.006 | 0.005 |
| *Phascolarctobacterium* | 0.007 | 0.007 | 0.005 |
| Clostridiales;Other;Other | 0.006 | 0.005 | 0.007 |
| *Erysipelotrichaceae* | 0.006 | 0.008 | 0.005 |
| *Lactobacillus* | 0.003 | 0.008 | 0.006 |
| *Sutterella* | 0.009 | 0.003 | 0.003 |

Cluster 1, n= 31; Cluster 2, n= 14; Cluster 3, n= 30. OTU= operational taxonomical unit.

Table S3: Results of the Mann Whitney U test of major bacterial genera (>1% of total OTUs) comparing clusters at baseline

| Bacteria | Cluster 1 vs 2 | Cluster 2 vs 3 | Cluster 1 vs 3 |
| --- | --- | --- | --- |
| *Bacteroides* | ****<0.0001 | 0.17 | <0.0001 |
| *Blautia* | 0.97 | 0.11 | **0.03 |
| *Lachnospiraceae* | 0.11 | 0.87 | 0.08 |
| *Faecalibacterium* | 0.99 | 0.23 | 0.17 |
| *Ruminococcaceae* | **0.004 | 0.12 | ****<0.0001 |
| *Prevotella* | ****<0.0001 | ****<0.0001 | 0.5 |
| *Coprococcus* | 0.16 | 0.49 | 0.01 |
| *Bifidobacterium* | 0.65 | 0.15 | 0.12 |
| *[Ruminococcus]* | *0.01 | 0.06 | 0.39 |
| *Ruminococcus* | 0.08 | ****<0.0001 | ****<0.0001 |
| *Dorea* | 0.06 | 0.49 | 0.26 |
| *Collinsella* | **0.005 | 0.06 | 0.2 |
| Clostridiales | 0.25 | 0.65 | 0.06 |
| *Streptococcus* | 0.47 | 0.68 | 0.75 |
| *Lachnospira* | 0.26 | 0.36 | 0.35 |
| *Megamonas* | 0.14 | **0.007 | 0.32 |
| *[Eubacterium]* | 0.46 | 0.78 | 0.74 |
| *Megasphaera* | 0.5 | 0.18 | *0.04 |
| *Oscillospira* | 0.38 | 0.95 | 0.39 |
| *Enterobacteriaceae* | 0.09 | *0.01 | 0.14 |
| *Parabacteroides* | 0.26 | 0.23 | ***0.009 |
| *Phascolarctobacterium* | 0.49 | 0.91 | 0.08 |
| Clostridiales;Other;Other | 0.41 | 0.29 | 0.96 |
| *Erysipelotrichaceae* | **0.04 | **0.02 | 0.84 |
| *Lactobacillus* | 0.23 | 0.18 | 0.33 |
| *Sutterella* | *0.01 | 0.4 | ****0.0005 |

Two-tailed p values derived from Mann Whitney U non-parametric test. The bacteria which were significantly different between each cluster are presented as **** p < 0.0001, *** p ≥ 0.0001 - < 0.001, ** p ≥ 0.001 - < 0.01, * p ≥ 0.01- 0.05. Cluster 1, n= 31; Cluster 2, n= 14; Cluster 3, n= 30. OTU= operational taxonomical unit.

Table S4: Frequency of dietary consumption per week at baseline (Mean ± SD)

| Foods | Cluster 1 | Cluster 2 | Cluster 3 |
| --- | --- | --- | --- |
| Carbohydrate-rich foods | 30.72 ± 12.19 | 18.38 ± 7.60 | 36.16 ± 11.38 |
| Protein-rich foods | 35.94 ± 13.68 | 23.98 ± 14.53 | 41.06 ± 18.04 |
| Vegetables | 5.22 ± 3.03 | 11.00 ± 4.57 | 5.53 ± 2.57 |
| Fruits | 3.61 ± 4.49 | 2.80 ± 2.14 | 4.28 ± 2.72 |
| Nuts | 4.11 ± 3.81 | 2.70 ± 3.71 | 4.13 ± 3.46 |
| Beverages | 16.11 ± 6.82 | 9.25 ± 13.02 | 15.06 ± 6.11 |
| the highest frequency of consumption (no. of food types) | 1 | 1 | 4 |
| the lowest frequency of consumption (no. of food types) | 1 | 5 | 0 |

SD= standard deviation.

Table S5: Results of the Mann Whitney U test of dietary consumption per week comparing clusters at baseline

| Foods | Cluster 1 vs 2 | Cluster 1 vs 3 | Cluster 2 vs 3 |
| --- | --- | --- | --- |
| Carbohydrate-rich foods | *0.018 | 0.282 | ***0.0005 |
| Protein-rich foods | 0.090 | 0.282 | *0.017 |
| Vegetables | **0.005 | 0.751 | ***0.0009 |
| Fruits | 0.990 | 0.212 | 0.185 |
| Nuts | 0.356 | 0.938 | 0.138 |
| Beverages | 0.082 | 0.513 | *0.042 |

Two-tailed p values derived from Mann Whitney U non-parametric test. The foods which were significantly different between each cluster are presented as *** p < 0.001, ** p ≥ 0.001 - < 0.01, * p ≥ 0.01- 0.05.

Table S6: Results of permutational multivariate analysis of variance (PERMANOVA) test comparing different clusters of major faecal microbiota and dietary consumption at baseline

| Pairs | Sums of squared | F.Model | R^2^ | p-value | p-adjusted |
| --- | --- | --- | --- | --- | --- |
| Cluster 1 vs Cluster 2 | 0.154 | 5.251 | 0.236 | **0.006 | *0.019 |
| Cluster 1 vs Cluster 3 | 0.038 | 1.937 | 0.078 | 0.096 | 0.287 |
| Cluster 2 vs Cluster 3 | 0.230 | 10.318 | 0.301 | ***0.0002 | ***0.0006 |

R^2^ and p values shown were calculated by Adonis test and 4999 permutations using the square root of Bray-Curtis distance of the relative abundances of major bacterial genera and frequency of food items consumption per week. p values were adjusted by Bonferroni’s multiple pairwise tests and presented as *** p < 0.001, ** p ≥ 0.001 - < 0.01, * p ≥ 0.01- 0.05.

Table S7: Results of permutational multivariate analysis of variance (PERMANOVA) test comparing races in different clusters of faecal microbiota at baseline

| Pairs | Sums of squared | F.Model | R^2^ | p-value | p-adjusted |
| --- | --- | --- | --- | --- | --- |
| Cluster 1 Chinese vs Cluster 2 Chinese | 0.349 | 6.952 | 0.17 | **0 | *0.01 |
| Cluster 1 Chinese vs Cluster 3 Chinese | 0.341 | 5.931 | 0.106 | **0 | *0.01 |
| Cluster 2 Chinese vs Cluster 3 Chinese | 0.241 | 4.142 | 0.115 | **0 | *0.01 |
| Cluster 1 Malay vs Cluster 2 Malay | 0.105 | 2.324 | 0.368 | 0.1 | 1 |
| Cluster 1 Malay vs Cluster 3 Malay | 0.135 | 3.607 | 0.546 | 0.1 | 1 |
| Cluster 2 Malay vs Cluster 3 Malay | 0.086 | 1.492 | 0.332 | 0.3 | 1 |
| Cluster 1 Indian vs Cluster 3 Indian | 0.107 | NA | 1 | NA | NA |
| Cluster 1 Chinese vs Cluster 1 Malay | 0.065 | 1.28 | 0.044 | 0.165 | 1 |
| Cluster 1 Chinese vs Cluster 1 Indian | 0.083 | 1.594 | 0.058 | 0.114 | 1 |
| Cluster 1 Malay vs Cluster 1 Indian | 0.089 | 2.935 | 0.595 | 0.25 | 1 |
| Cluster 2 Chinese vs Cluster 2 Malay | 0.055 | 1.176 | 0.105 | 0.268 | 1 |
| Cluster 2 Chinese vs Cluster 2 Korean | 0.063 | 1.467 | 0.155 | 0.105 | 1 |
| Cluster 2 Chinese vs Cluster 2 Vietnamese | 0.043 | 0.988 | 0.11 | 0.601 | 1 |
| Cluster 2 Malay vs Cluster 2 Korean | 0.062 | 1.029 | 0.34 | 0.5 | 1 |
| Cluster 2 Malay vs Cluster 2 Vietnamese | 0.039 | 0.646 | 0.244 | 1 | 1 |
| Cluster 2 Korean vs Cluster 2 Vietnamese | 0.044 | NA | 1 | NA | NA |
| Cluster 3 Chinese vs Cluster 3 Malay | 0.136 | 2.165 | 0.08 | *0.034 | 1 |
| Cluster 3 Chinese vs Cluster 3 Indian | 0.043 | 0.683 | 0.028 | 0.612 | 1 |
| Cluster 3 Chinese vs Cluster 3 Caucasian | 0.075 | 1.189 | 0.047 | 0.293 | 1 |
| Cluster 3 Chinese vs Cluster 3 Filipino | 0.048 | 0.755 | 0.03 | 0.617 | 1 |
| Cluster 3 Malay vs Cluster 3 Indian | 0.074 | 1.424 | 0.588 | 0.333 | 1 |
| Cluster 3 Malay vs Cluster 3 Caucasian | 0.105 | 2.029 | 0.67 | 0.333 | 1 |
| Cluster 3 Malay vs Cluster 3 Filipino | 0.069 | 1.335 | 0.572 | 0.667 | 1 |
| Cluster 3 Indian vs Cluster 3 Caucasian | 0.068 | NA | 1 | NA | NA |
| Cluster 3 Indian vs Cluster 3 Filipino | 0.057 | NA | 1 | NA | NA |
| Cluster 3 Caucasian vs Cluster 3 Filipino | 0.036 | NA | 1 | NA | NA |

R^2^ and p values shown were calculated by Adonis test and 4999 permutations using the square root of Bray-Curtis distance of the relative abundances of bacterial genera. p values were adjusted by Bonferroni’s multiple pairwise tests and presented as ** p < 0.01, * p ≥ 0.01- 0.05. NA= not available. Cluster 1 Chinese: n= 27, Cluster 1 Malay: n= 3, Cluster 1 Indian: n= 1, Cluster 2 Chinese: n= 9, Cluster 2 Malay: n= 3, Cluster 2 Korean: n=1, Cluster 2 Vietnamese: n= 1, Cluster 3 Chinese: n= 25, Cluster 3 Malay: n= 2, Cluster 3 Indian: n= 1, Cluster 3 Caucasian: n= 1, Cluster 3 Filipino: n= 1.

Table S8: Results of permutational multivariate analysis of variance (PERMANOVA) test comparing genders in different clusters of faecal microbiota for all time points

| Time points | Pairs | Sums of Squared | F Model | R^2^ | p-values | p-adjusted |
| --- | --- | --- | --- | --- | --- | --- |
| Timepoint 1 | Cluster1 Female vs Cluster1 Male | 0.034 | 0.639 | 0.022 | 0.898 | 1 |
|  | Cluster2 Male vs Cluster2 Female | 0.073 | 1.582 | 0.116 | 0.044 | 0.66 |
|  | Cluster3 Female vs Cluster3 Male | 0.095 | 1.497 | 0.051 | 0.076 | 1 |
|  | Cluster1 Female vs Cluster2 Female | 0.309 | 6.162 | 0.192 | 0 | **0.003 |
|  | Cluster1 Female vs Cluster3 Female | 0.294 | 5.519 | 0.133 | 0 | **0.003 |
|  | Cluster2 Male vs Cluster3 Female | 0.201 | 3.783 | 0.147 | 0 | **0.003 |
|  | Cluster1 Male vs Cluster2 Male | 0.248 | 4.76 | 0.241 | 0 | **0.003 |
|  | Cluster1 Male vs Cluster3 Male | 0.191 | 2.879 | 0.121 | 0.001 | *0.012 |
|  | Cluster2 Male vs Cluster3 Male | 0.188 | 2.754 | 0.133 | 0.001 | **0.009 |
| Timepoint 2 | Cluster1 Female vs Cluster1 Male | 0.045 | 0.748 | 0.025 | 0.777 | 1 |
|  | Cluster2 Male vs Cluster2 Female | 0.082 | 1.243 | 0.094 | 0.2 | 1 |
|  | Cluster3 Female vs Cluster3 Male | 0.099 | 1.798 | 0.06 | 0.026 | 0.39 |
|  | Cluster1 Female vs Cluster2 Female | 0.192 | 2.787 | 0.097 | 0.003 | 0.051 |
|  | Cluster1 Female vs Cluster3 Female | 0.284 | 4.577 | 0.113 | 0 | **0.003 |
|  | Cluster2 Female vs Cluster3 Female | 0.126 | 2.002 | 0.083 | 0.034 | 0.516 |
|  | Cluster1 Male vs Cluster2 Male | 0.183 | 3.69 | 0.197 | 0 | **0.003 |
|  | Cluster1 Male vs Cluster3 Male | 0.146 | 2.895 | 0.121 | 0.003 | *0.045 |
|  | Cluster2 Male vs Cluster3 Male | 0.174 | 3.314 | 0.156 | 0.001 | **0.009 |
| Timepoint 3 | Cluster1 Female vs Cluster1 Male | 0.055 | 0.953 | 0.032 | 0.527 | 1 |
|  | Cluster2 Male vs Cluster2 Female | 0.082 | 1.283 | 0.097 | 0.187 | 1 |
|  | Cluster3 Female vs Cluster3 Male | 0.11 | 2.033 | 0.068 | 0.009 | 0.132 |
|  | Cluster1 Female vs Cluster2 Female | 0.195 | 3.248 | 0.111 | 0 | **0.006 |
|  | Cluster1 Female vs Cluster3 Female | 0.222 | 3.947 | 0.099 | 0 | **0.003 |
|  | Cluster2 Female vs Cluster3 Female | 0.102 | 1.831 | 0.077 | 0.031 | 0.465 |
|  | Cluster1 Male vs Cluster2 Male | 0.192 | 3.272 | 0.179 | 0 | **0.006 |
|  | Cluster1 Male vs Cluster3 Male | 0.118 | 2.121 | 0.092 | 0.008 | 0.114 |
|  | Cluster2 Male vs Cluster3 Male | 0.195 | 3.304 | 0.155 | 0.001 | *0.012 |
| Timepoint 4 | Cluster1 Female vs Cluster1 Male | 0.041 | 0.657 | 0.022 | 0.873 | 1 |
|  | Cluster2 Male vs Cluster2 Female | 0.065 | 0.768 | 0.06 | 0.694 | 1 |
|  | Cluster3 Female vs Cluster3 Male | 0.086 | 1.172 | 0.04 | 0.263 | 1 |
|  | Cluster1 Female vs Cluster2 Female | 0.159 | 2.21 | 0.078 | 0.01 | 0.144 |
|  | Cluster1 Female vs Cluster3 Female | 0.18 | 2.433 | 0.063 | 0.001 | *0.018 |
|  | Cluster2 Female vs Cluster3 Female | 0.162 | 1.905 | 0.08 | 0.027 | 0.402 |
|  | Cluster1 Male vs Cluster2 Male | 0.117 | 1.809 | 0.108 | 0.021 | 0.309 |
|  | Cluster1 Male vs Cluster3 Male | 0.128 | 2.224 | 0.096 | 0.003 | *0.042 |
|  | Cluster2 Male vs Cluster3 Male | 0.082 | 1.234 | 0.064 | 0.194 | 1 |

R^2^ and p values shown were calculated by Adonis test and 4999 permutations using the square root of Bray-Curtis distance of the relative abundances of bacterial genera. p values were adjusted by Bonferroni’s multiple pairwise tests and presented as ** p < 0.01, * p ≥ 0.01- 0.05. Cluster 1 Female: n= 21, Cluster 1 Male: n= 10, Cluster 2 Female: n= 7, Cluster 2 Male: n= 7, Cluster 3 Female: n= 17, Cluster 3 Male: n= 13.

Table S9: Results of permutational multivariate analysis of variance (PERMANOVA) test comparing types of Bristol stool scale in different clusters of major faecal microbiota at baseline

| Pairs | Sums of squared | F.Model | R2 | p-value | p-adjusted |
| --- | --- | --- | --- | --- | --- |
| Cluster1 Type2 vs Cluster1 Type1 | 0.029 | 0.657 | 0.141 | 0.9 | 1 |
| Cluster1 Type2 vs Cluster1 Type5 | 0.031 | 0.684 | 0.255 | 0.75 | 1 |
| Cluster1 Type3 vs Cluster1 Type1 | 0.052 | 0.985 | 0.099 | 0.468 | 1 |
| Cluster1 Type3 vs Cluster1 Type2 | 0.04 | 0.758 | 0.078 | 0.789 | 1 |
| Cluster1 Type3 vs Cluster1 Type5 | 0.038 | 0.68 | 0.089 | 0.776 | 1 |
| Cluster1 Type4 vs Cluster1 Type1 | 0.05 | 0.917 | 0.051 | 0.546 | 1 |
| Cluster1 Type4 vs Cluster1 Type2 | 0.04 | 0.734 | 0.041 | 0.784 | 1 |
| Cluster1 Type4 vs Cluster1 Type3 | 0.042 | 0.751 | 0.033 | 0.758 | 1 |
| Cluster1 Type4 vs Cluster1 Type5 | 0.033 | 0.595 | 0.038 | 0.937 | 1 |
| Cluster1 Type5 vs Cluster1 Type1 | 0.033 | 0.771 | 0.278 | 0.75 | 1 |
| Cluster2 Type4 vs Cluster2 Type3 | 0.029 | 0.57 | 0.06 | 0.934 | 1 |
| Cluster2 Type5 vs Cluster2 Type3 | 0.036 | 0.753 | 0.131 | 0.829 | 1 |
| Cluster2 Type5 vs Cluster2 Type4 | 0.038 | 0.751 | 0.086 | 0.844 | 1 |
| Cluster3 Type2 vs Cluster3 Type1 | 0.052 | 0.929 | 0.188 | 0.533 | 1 |
| Cluster3 Type2 vs Cluster3 Type3 | 0.075 | 1.34 | 0.143 | 0.138 | 1 |
| Cluster3 Type2 vs Cluster3 Type4 | 0.085 | 1.366 | 0.079 | 0.152 | 1 |
| Cluster3 Type2 vs Cluster3 Type5 | 0.087 | 1.603 | 0.243 | 0.172 | 1 |
| Cluster3 Type2 vs Cluster3 Type6 | 0.143 | 2.655 | 0.47 | 0.2 | 1 |
| Cluster3 Type3 vs Cluster3 Type1 | 0.067 | 1.149 | 0.161 | 0.296 | 1 |
| Cluster3 Type3 vs Cluster3 Type4 | 0.061 | 0.982 | 0.052 | 0.457 | 1 |
| Cluster3 Type3 vs Cluster3 Type6 | 0.137 | 2.383 | 0.323 | 0.143 | 1 |
| Cluster3 Type4 vs Cluster3 Type1 | 0.041 | 0.639 | 0.044 | 0.822 | 1 |
| Cluster3 Type4 vs Cluster3 Type6 | 0.144 | 2.262 | 0.148 | 0.128 | 1 |
| Cluster3 Type5 vs Cluster3 Type1 | 0.052 | 0.91 | 0.233 | 0.5 | 1 |
| Cluster3 Type5 vs Cluster3 Type3 | 0.062 | 1.085 | 0.134 | 0.393 | 1 |
| Cluster3 Type5 vs Cluster3 Type4 | 0.083 | 1.331 | 0.081 | 0.177 | 1 |
| Cluster3 Type5 vs Cluster3 Type6 | 0.138 | 2.54 | 0.559 | 0.25 | 1 |
| Cluster3 Type6 vs Cluster3 Type1 | 0.118 | 1.939 | 0.66 | 0.333 | 1 |
| Cluster1 Type1 vs Cluster3 Type1 | 0.089 | 1.816 | 0.377 | 0.1 | 1 |
| Cluster1 Type2 vs Cluster3 Type2 | 0.066 | 1.313 | 0.208 | 0.171 | 1 |
| Cluster1 Type3 vs Cluster2 Type3 | 0.165 | 3.085 | 0.236 | **0.001 | 0.09 |
| Cluster1 Type3 vs Cluster3 Type3 | 0.12 | 2.122 | 0.15 | **0.008 | 0.72 |
| Cluster1 Type4 vs Cluster2 Type4 | 0.277 | 5.041 | 0.194 | **0 | *0.018 |
| Cluster1 Type4 vs Cluster3 Type4 | 0.303 | 5.087 | 0.154 | **0 | *0.018 |
| Cluster1 Type5 vs Cluster2 Type5 | 0.079 | 1.703 | 0.46 | 0.25 | 1 |
| Cluster1 Type5 vs Cluster3 Type5 | 0.061 | 1.111 | 0.357 | 0.5 | 1 |
| Cluster2 Type3 vs Cluster3 Type3 | 0.122 | 2.234 | 0.218 | **0.005 | 0.45 |
| Cluster2 Type4 vs Cluster3 Type4 | 0.154 | 2.554 | 0.118 | **0.003 | 0.306 |
| Cluster2 Type5 vs Cluster3 Type5 | 0.113 | 2.242 | 0.359 | 0.1 | 1 |

R^2^ and p values shown were calculated by Adonis test and 4999 permutations using the square root of Bray-Curtis distance of the relative abundances of bacterial genera. p values were adjusted by Bonferroni’s multiple pairwise tests and presented as *** p ≥ 0.0001 - < 0.001, ** p ≥ 0.001 - < 0.01, * p ≥ 0.01- 0.05.

Table S10: Results of permutational multivariate analysis of variance (PERMANOVA) test comparing types of Bristol stool scale in different clusters of major faecal microbiota at Timepoint 2

| Pairs | Sums of squared | F.Model | R2 | | p-value | p-adjusted |
| --- | --- | --- | --- | --- | --- | --- |
| Cluster1 Type2 vs Cluster1 Type1 | 0.049 | 0.918 | 0.187 | 0.5 | | 1 |
| Cluster1 Type2 vs Cluster1 Type6 | 0.03 | 0.556 | 0.122 | 0.667 | | 1 |
| Cluster1 Type3 vs Cluster1 Type1 | 0.044 | 0.62 | 0.049 | 1 | | 1 |
| Cluster1 Type3 vs Cluster1 Type2 | 0.039 | 0.587 | 0.035 | 0.898 | | 1 |
| Cluster1 Type3 vs Cluster1 Type4 | 0.046 | 0.702 | 0.032 | 0.814 | | 1 |
| Cluster1 Type3 vs Cluster1 Type5 | 0.033 | 0.472 | 0.038 | 1 | | 1 |
| Cluster1 Type3 vs Cluster1 Type6 | 0.047 | 0.658 | 0.052 | 0.929 | | 1 |
| Cluster1 Type4 vs Cluster1 Type1 | 0.062 | 1.075 | 0.107 | 0.363 | | 1 |
| Cluster1 Type4 vs Cluster1 Type2 | 0.043 | 0.764 | 0.056 | 0.758 | | 1 |
| Cluster1 Type4 vs Cluster1 Type6 | 0.043 | 0.741 | 0.076 | 0.551 | | 1 |
| Cluster1 Type5 vs Cluster1 Type1 | 0.039 | NA | 1 | NA | | NA |
| Cluster1 Type5 vs Cluster1 Type2 | 0.026 | 0.482 | 0.108 | 1 | | 1 |
| Cluster1 Type5 vs Cluster1 Type4 | 0.028 | 0.489 | 0.052 | 1 | | 1 |
| Cluster1 Type5 vs Cluster1 Type6 | 0.025 | NA | 1 | NA | | NA |
| Cluster1 Type6 vs Cluster1 Type1 | 0.055 | NA | 1 | NA | | NA |
| Cluster2 Type3 vs Cluster2 Type5 | 0.095 | 2.095 | 0.512 | 0.333 | | 1 |
| Cluster2 Type4 vs Cluster2 Type3 | 0.064 | 0.95 | 0.087 | 0.38 | | 1 |
| Cluster2 Type4 vs Cluster2 Type5 | 0.068 | 0.989 | 0.09 | 0.339 | | 1 |
| Cluster3 Type2 vs Cluster3 Type1 | 0.055 | 1.003 | 0.334 | 0.5 | | 1 |
| Cluster3 Type2 vs Cluster3 Type6 | 0.09 | 1.568 | 0.343 | 0.2 | | 1 |
| Cluster3 Type3 vs Cluster3 Type1 | 0.036 | 0.665 | 0.117 | 0.714 | | 1 |
| Cluster3 Type3 vs Cluster3 Type2 | 0.09 | 1.665 | 0.192 | 0.052 | | 1 |
| Cluster3 Type3 vs Cluster3 Type4 | 0.093 | 1.663 | 0.1 | *0.048 | | 1 |
| Cluster3 Type3 vs Cluster3 Type5 | 0.062 | 1.29 | 0.105 | 0.175 | | 1 |
| Cluster3 Type3 vs Cluster3 Type6 | 0.109 | 1.964 | 0.247 | 0.081 | | 1 |
| Cluster3 Type4 vs Cluster3 Type1 | 0.031 | 0.553 | 0.052 | 1 | | 1 |
| Cluster3 Type4 vs Cluster3 Type2 | 0.069 | 1.22 | 0.092 | 0.255 | | 1 |
| Cluster3 Type4 vs Cluster3 Type6 | 0.124 | 2.169 | 0.165 | *0.026 | | 1 |
| Cluster3 Type5 vs Cluster3 Type1 | 0.019 | 0.456 | 0.071 | 1 | | 1 |
| Cluster3 Type5 vs Cluster3 Type2 | 0.092 | 2.006 | 0.2 | *0.017 | | 1 |
| Cluster3 Type5 vs Cluster3 Type4 | 0.065 | 1.258 | 0.073 | 0.218 | | 1 |
| Cluster3 Type5 vs Cluster3 Type6 | 0.082 | 1.809 | 0.205 | 0.087 | | 1 |
| Cluster3 Type6 vs Cluster3 Type1 | 0.059 | 0.941 | 0.485 | 0.667 | | 1 |
| Cluster1 Type1 vs Cluster3 Type1 | 0.056 | NA | 1 | NA | | NA |
| Cluster1 Type2 vs Cluster3 Type2 | 0.098 | 1.819 | 0.233 | 0.11 | | 1 |
| Cluster1 Type3 vs Cluster2 Type3 | 0.142 | 2.082 | 0.138 | *0.04 | | 1 |
| Cluster1 Type3 vs Cluster3 Type3 | 0.121 | 1.84 | 0.098 | *0.031 | | 1 |
| Cluster1 Type4 vs Cluster2 Type4 | 0.172 | 2.663 | 0.129 | **0.008 | | 0.792 |
| Cluster1 Type4 vs Cluster3 Type4 | 0.137 | 2.391 | 0.112 | **0.002 | | 0.238 |
| Cluster1 Type5 vs Cluster2 Type5 | 0.095 | 1.799 | 0.643 | 0.333 | | 1 |
| Cluster1 Type5 vs Cluster3 Type5 | 0.055 | 1.298 | 0.178 | 0.254 | | 1 |
| Cluster1 Type6 vs Cluster3 Type6 | 0.054 | 0.863 | 0.463 | 0.667 | | 1 |
| Cluster2 Type3 vs Cluster3 Type3 | 0.16 | 3.115 | 0.342 | *0.03 | | 1 |
| Cluster2 Type4 vs Cluster3 Type4 | 0.132 | 2.081 | 0.099 | *0.024 | | 1 |
| Cluster2 Type5 vs Cluster3 Type5 | 0.094 | 2.135 | 0.234 | *0.029 | | 1 |

R^2^ and p values shown were calculated by Adonis test and 4999 permutations using the square root of Bray-Curtis distance of the relative abundances of bacterial genera. p values were adjusted by Bonferroni’s multiple pairwise tests and presented as ** p < 0.01, * p ≥ 0.01- 0.05. NA= not available.

Table S11: Results of permutational multivariate analysis of variance (PERMANOVA) test comparing types of Bristol stool scale in different clusters of major faecal microbiota at Timepoint 3

| Pairs | Sums of squared | F.Model | R2 | p-value | p-adjusted |
| --- | --- | --- | --- | --- | --- |
| Cluster1 Type2 vs Cluster1 Type4 | 0.044 | 0.8 | 0.04 | 0.763 | 1 |
| Cluster1 Type3 vs Cluster1 Type2 | 0.075 | 1.272 | 0.089 | 0.199 | 1 |
| Cluster1 Type3 vs Cluster1 Type4 | 0.076 | 1.19 | 0.056 | 0.247 | 1 |
| Cluster1 Type3 vs Cluster1 Type5 | 0.045 | 0.623 | 0.082 | 1 | 1 |
| Cluster1 Type3 vs Cluster1 Type6 | 0.034 | 0.469 | 0.063 | 1 | 1 |
| Cluster1 Type5 vs Cluster1 Type2 | 0.028 | 0.627 | 0.095 | 0.748 | 1 |
| Cluster1 Type5 vs Cluster1 Type4 | 0.044 | 0.737 | 0.054 | 0.8 | 1 |
| Cluster1 Type6 vs Cluster1 Type2 | 0.029 | 0.669 | 0.1 | 0.627 | 1 |
| Cluster1 Type6 vs Cluster1 Type4 | 0.03 | 0.508 | 0.038 | 1 | 1 |
| Cluster1 Type6 vs Cluster1 Type5 | 0.035 | NA | 1 | NA | NA |
| Cluster2 Type2 vs Cluster2 Type3 | 0.086 | NA | 1 | NA | NA |
| Cluster2 Type4 vs Cluster2 Type2 | 0.113 | 3.016 | 0.335 | 0.118 | 1 |
| Cluster2 Type4 vs Cluster2 Type3 | 0.137 | 3.649 | 0.378 | 0.122 | 1 |
| Cluster2 Type4 vs Cluster2 Type5 | 0.041 | 0.915 | 0.103 | 0.612 | 1 |
| Cluster2 Type4 vs Cluster2 Type6 | 0.031 | 0.837 | 0.122 | 0.752 | 1 |
| Cluster2 Type4 vs Cluster2 Type7 | 0.147 | 3.925 | 0.395 | 0.117 | 1 |
| Cluster2 Type5 vs Cluster2 Type2 | 0.1 | 1.462 | 0.422 | 0.5 | 1 |
| Cluster2 Type5 vs Cluster2 Type3 | 0.124 | 1.815 | 0.476 | 0.25 | 1 |
| Cluster2 Type5 vs Cluster2 Type6 | 0.051 | 0.741 | 0.27 | 0.75 | 1 |
| Cluster2 Type5 vs Cluster2 Type7 | 0.156 | 2.285 | 0.533 | 0.25 | 1 |
| Cluster2 Type6 vs Cluster2 Type2 | 0.061 | NA | 1 | NA | NA |
| Cluster2 Type6 vs Cluster2 Type3 | 0.086 | NA | 1 | NA | NA |
| Cluster2 Type6 vs Cluster2 Type7 | 0.082 | NA | 1 | NA | NA |
| Cluster2 Type7 vs Cluster2 Type2 | 0.158 | NA | 1 | NA | NA |
| Cluster2 Type7 vs Cluster2 Type3 | 0.172 | NA | 1 | NA | NA |
| Cluster3 Type3 vs Cluster3 Type2 | 0.038 | 0.616 | 0.072 | 0.843 | 1 |
| Cluster3 Type3 vs Cluster3 Type4 | 0.037 | 0.673 | 0.038 | 0.851 | 1 |
| Cluster3 Type3 vs Cluster3 Type5 | 0.022 | 0.407 | 0.043 | 0.989 | 1 |
| Cluster3 Type3 vs Cluster3 Type6 | 0.077 | 1.339 | 0.182 | 0.22 | 1 |
| Cluster3 Type4 vs Cluster3 Type2 | 0.053 | 0.911 | 0.057 | 0.551 | 1 |
| Cluster3 Type4 vs Cluster3 Type5 | 0.036 | 0.659 | 0.04 | 0.888 | 1 |
| Cluster3 Type4 vs Cluster3 Type6 | 0.078 | 1.4 | 0.097 | 0.163 | 1 |
| Cluster3 Type5 vs Cluster3 Type2 | 0.058 | 0.968 | 0.122 | 0.545 | 1 |
| Cluster3 Type5 vs Cluster3 Type6 | 0.078 | 1.42 | 0.221 | 0.142 | 1 |
| Cluster3 Type6 vs Cluster3 Type2 | 0.058 | 0.855 | 0.176 | 0.467 | 1 |
| Cluster1 Type2 vs Cluster2 Type2 | 0.072 | 1.633 | 0.214 | 0.242 | 1 |
| Cluster1 Type2 vs Cluster3 Type2 | 0.082 | 1.569 | 0.148 | 0.089 | 1 |
| Cluster1 Type3 vs Cluster2 Type3 | 0.054 | 0.757 | 0.098 | 0.882 | 1 |
| Cluster1 Type3 vs Cluster3 Type3 | 0.111 | 1.696 | 0.124 | 0.053 | 1 |
| Cluster1 Type4 vs Cluster2 Type4 | 0.228 | 4.357 | 0.187 | **0 | *0.021 |
| Cluster1 Type4 vs Cluster3 Type4 | 0.138 | 2.4 | 0.088 | **0 | *0.042 |
| Cluster1 Type5 vs Cluster2 Type5 | 0.079 | 1.154 | 0.366 | 0.5 | 1 |
| Cluster1 Type5 vs Cluster3 Type5 | 0.05 | 0.958 | 0.193 | 0.5 | 1 |
| Cluster1 Type6 vs Cluster2 Type6 | 0.068 | NA | 1 | NA | NA |
| Cluster1 Type6 vs Cluster3 Type6 | 0.053 | 0.837 | 0.456 | 0.667 | 1 |
| Cluster2 Type2 vs Cluster3 Type2 | 0.072 | 1.032 | 0.256 | 0.6 | 1 |
| Cluster2 Type3 vs Cluster3 Type3 | 0.101 | 1.788 | 0.263 | 0.142 | 1 |
| Cluster2 Type4 vs Cluster3 Type4 | 0.136 | 2.763 | 0.133 | **0.002 | 0.21 |
| Cluster2 Type5 vs Cluster3 Type5 | 0.086 | 1.488 | 0.199 | 0.124 | 1 |
| Cluster2 Type6 vs Cluster3 Type6 | 0.062 | 0.99 | 0.497 | 0.667 | 1 |

R^2^ and p values shown were calculated by Adonis test and 4999 permutations using the square root of Bray-Curtis distance of the relative abundances of bacterial genera. p values were adjusted by Bonferroni’s multiple pairwise tests and presented as ** p < 0.01, * p ≥ 0.01- 0.05.

Table S12: Results of permutational multivariate analysis of variance (PERMANOVA) test comparing types of Bristol stool scale in different clusters of major faecal microbiota at Timepoint 4

| Pairs | Sums of squared | F.Model | R2 | p-value | p-adjusted |
| --- | --- | --- | --- | --- | --- |
| Cluster1 Type2 vs Cluster1 Type1 | 0.092 | 1.399 | 0.318 | 0.200 | 1 |
| Cluster1 Type3 vs Cluster1 Type1 | 0.085 | 1.906 | 0.175 | 0.054 | 1 |
| Cluster1 Type3 vs Cluster1 Type2 | 0.073 | 1.478 | 0.129 | 0.165 | 1 |
| Cluster1 Type4 vs Cluster1 Type1 | 0.095 | 1.296 | 0.085 | 0.200 | 1 |
| Cluster1 Type4 vs Cluster1 Type2 | 0.053 | 0.714 | 0.045 | 0.794 | 1 |
| Cluster1 Type4 vs Cluster1 Type3 | 0.077 | 1.231 | 0.055 | 0.224 | 1 |
| Cluster1 Type4 vs Cluster1 Type5 | 0.029 | 0.407 | 0.026 | 0.991 | 1 |
| Cluster1 Type5 vs Cluster1 Type1 | 0.079 | 1.730 | 0.366 | 0.100 | 1 |
| Cluster1 Type5 vs Cluster1 Type2 | 0.064 | 1.112 | 0.218 | 0.300 | 1 |
| Cluster1 Type5 vs Cluster1 Type3 | 0.038 | 0.878 | 0.081 | 0.605 | 1 |
| Cluster2 Type4 vs Cluster2 Type2 | 0.044 | 0.544 | 0.052 | 0.833 | 1 |
| Cluster2 Type4 vs Cluster2 Type5 | 0.137 | 1.674 | 0.132 | 0.104 | 1 |
| Cluster2 Type5 vs Cluster2 Type2 | 0.089 | 0.925 | 0.480 | 0.667 | 1 |
| Cluster3 Type2 vs Cluster3 Type1 | 0.115 | 0.345 | 0.257 | 1.000 | 1 |
| Cluster3 Type3 vs Cluster3 Type1 | 0.052 | 0.761 | 0.202 | 0.800 | 1 |
| Cluster3 Type3 vs Cluster3 Type2 | 0.188 | 1.400 | 0.259 | 0.333 | 1 |
| Cluster3 Type3 vs Cluster3 Type4 | 0.084 | 1.543 | 0.088 | 0.074 | 1 |
| Cluster3 Type3 vs Cluster3 Type5 | 0.084 | 1.491 | 0.142 | 0.101 | 1 |
| Cluster3 Type3 vs Cluster3 Type6 | 0.066 | 0.892 | 0.182 | 0.733 | 1 |
| Cluster3 Type4 vs Cluster3 Type1 | 0.099 | 1.943 | 0.130 | 0.068 | 1 |
| Cluster3 Type4 vs Cluster3 Type2 | 0.247 | 3.460 | 0.198 | 0.126 | 1 |
| Cluster3 Type4 vs Cluster3 Type6 | 0.059 | 1.086 | 0.072 | 0.290 | 1 |
| Cluster3 Type5 vs Cluster3 Type1 | 0.098 | 1.923 | 0.243 | 0.247 | 1 |
| Cluster3 Type5 vs Cluster3 Type2 | 0.219 | 2.401 | 0.255 | 0.198 | 1 |
| Cluster3 Type5 vs Cluster3 Type4 | 0.051 | 0.993 | 0.050 | 0.461 | 1 |
| Cluster3 Type5 vs Cluster3 Type6 | 0.057 | 0.997 | 0.125 | 0.441 | 1 |
| Cluster3 Type6 vs Cluster3 Type1 | 0.078 | 0.833 | 0.454 | 1.000 | 1 |
| Cluster3 Type6 vs Cluster3 Type2 | 0.124 | 0.581 | 0.225 | 1.000 | 1 |
| Cluster1 Type1 vs Cluster3 Type1 | 0.062 | 1.185 | 0.542 | 0.667 | 1 |
| Cluster1 Type2 vs Cluster2 Type2 | 0.040 | 0.550 | 0.216 | 1.000 | 1 |
| Cluster1 Type2 vs Cluster3 Type2 | 0.195 | 1.226 | 0.290 | 0.300 | 1 |
| Cluster1 Type3 vs Cluster3 Type3 | 0.060 | 1.194 | 0.098 | 0.258 | 1 |
| Cluster1 Type4 vs Cluster2 Type4 | 0.153 | 1.981 | 0.079 | *0.022 | 1 |
| Cluster1 Type4 vs Cluster3 Type4 | 0.256 | 4.076 | 0.136 | **0 | *0.036 |
| Cluster1 Type5 vs Cluster2 Type5 | 0.180 | 2.997 | 0.500 | 0.100 | 1 |
| Cluster1 Type5 vs Cluster3 Type5 | 0.095 | 1.942 | 0.195 | 0.039 | 1 |
| Cluster2 Type2 vs Cluster3 Type2 | 0.128 | 0.386 | 0.279 | 1.000 | 1 |
| Cluster2 Type4 vs Cluster3 Type4 | 0.114 | 1.787 | 0.072 | 0.034 | 1 |
| Cluster2 Type5 vs Cluster3 Type5 | 0.179 | 3.124 | 0.309 | 0.029 | 1 |

R^2^ and p values shown were calculated by Adonis test and 4999 permutations using the square root of Bray-Curtis distance of the relative abundances of bacterial genera. p values were adjusted by Bonferroni’s multiple pairwise tests and presented as ** p < 0.01, * p ≥ 0.01- 0.05.

Table S13: Results of the one-way ANOVA and post-hoc Bonferroni’s multiple comparisons tests of alpha diversity comparing different clusters at baseline

| Pairs | P value (ANOVA) | | Adjusted P value (Bonferroni's multiple comparisons test) | |
| --- | --- | --- | --- | --- |
|  | Chao 1 | Shannon | Chao 1 | Shannon |
| Cluster 1_TP 1 vs. Cluster 1_TP 2 | 0.542 | 0.232 | >0.9999 | 0.787 |
| Cluster 1_TP 1 vs. Cluster 1_TP 3 |  |  | 0.924 | 0.314 |
| Cluster 1_TP 1 vs. Cluster 1_TP 4 |  |  | >0.9999 | >0.9999 |

P value and adjusted p values derived from the analysis of variances (ANOVA) and Bonferroni’s multiple pairwise comparisons tests. The Chao 1 and Shannon’s indexes were not significantly different between each cluster. TP= Timepoint. Cluster 1_TP1, _TP2, _TP3, _TP4 (n= 31, 31, 31, 31), Cluster 2_TP1, _TP2, _TP3, _TP4 (n= 14, 14, 14, 14): Cluster 3_TP1, _TP2, _TP3, _TP4 (n= 29, 30, 30, 27).

Table S14: Results of the one-way ANOVA and post-hoc Bonferroni’s multiple comparisons tests of alpha diversity comparing different time points within each cluster

| Pairs | P value (ANOVA) | | | Adjusted P value (Bonferroni's multiple comparisons test) | |
| --- | --- | --- | --- | --- | --- |
|  | Chao 1 | | Shannon | Chao 1 | Shannon |
| Cluster 1_TP 1 vs. Cluster 1_TP 2 | **0.006 | 0.545 | | >0.9999 | >0.9999 |
| Cluster 1_TP 1 vs. Cluster 1_TP 3 |  |  |  | 0.2401 | >0.9999 |
| Cluster 1_TP 1 vs. Cluster 1_TP 4 |  |  |  | 0.143 | >0.9999 |
| Cluster 1_TP 2 vs. Cluster 1_TP 3 |  |  |  | *0.014 | >0.9999 |
| Cluster 1_TP 2 vs. Cluster 1_TP 4 |  |  |  | 0.056 | >0.9999 |
| Cluster 1_TP 3 vs. Cluster 1_TP 4 |  |  |  | >0.9999 | >0.9999 |
| Cluster 2_TP 1 vs. Cluster 2_TP 2 | 0.245 | 0.653 | | >0.9999 | >0.9999 |
| Cluster 2_TP 1 vs. Cluster 2_TP 3 |  |  |  | >0.9999 | >0.9999 |
| Cluster 2_TP 1 vs. Cluster 2_TP 4 |  |  |  | >0.9999 | >0.9999 |
| Cluster 2_TP 2 vs. Cluster 2_TP 3 |  |  |  | 0.487 | >0.9999 |
| Cluster 2_TP 2 vs. Cluster 2_TP 4 |  |  |  | 0.686 | >0.9999 |
| Cluster 2_TP 3 vs. Cluster 2_TP 4 |  |  |  | >0.9999 | >0.9999 |
| Cluster 3_TP 1 vs. Cluster 3_TP 2 | **0.004 | 0.551 | | >0.9999 | >0.9999 |
| Cluster 3_TP 1 vs. Cluster 3_TP 3 |  |  |  | 0.395 | >0.9999 |
| Cluster 3_TP 1 vs. Cluster 3_TP 4 |  |  |  | 0.054 | >0.9999 |
| Cluster 3_TP 2 vs. Cluster 3_TP 3 |  |  |  | 0.446 | >0.9999 |
| Cluster 3_TP 2 vs. Cluster 3_TP 4 |  |  |  | 0.080 | >0.9999 |
| Cluster 3_TP 3 vs. Cluster 3_TP 4 |  |  |  | 0.660 | >0.9999 |

P value and adjusted p values derived from the analysis of variances (ANOVA) and Bonferroni’s multiple pairwise comparisons tests. The Chao 1 and Shannon’s indexes which were significantly different between time points in each cluster are presented as ** p < 0.01, * p ≥ 0.01- 0.05. TP= Timepoint. Cluster 1_TP1, _TP2, _TP3, _TP4 (n= 31, 31, 31, 31), Cluster 2_TP1, _TP2, _TP3, _TP4 (n= 14, 14, 14, 14): Cluster 3_TP1, _TP2, _TP3, _TP4 (n= 29, 30, 30, 27).

Table S15: Results of permutational multivariate analysis of variance (PERMANOVA) test of beta diversity comparing different time points within each cluster

| Cluster | Beta diversity | Pairs | Sums of squared | F.Model | R^2^ | p-value | p-adjusted |
| --- | --- | --- | --- | --- | --- | --- | --- |
| Cluster 1 | Unweighted Unifrac | TP1 vs TP2 | 0.849 | 4.302 | 0.067 | ***0.0002 | **0.0012 |
|  |  | TP1 vs TP3 | 0.839 | 4.257 | 0.066 | ***0.0002 | **0.0012 |
|  |  | TP1 vs TP4 | 0.71 | 3.468 | 0.055 | ***0.0002 | **0.0012 |
|  |  | TP2 vs TP3 | 0.757 | 4.054 | 0.063 | ***0.0002 | **0.0012 |
|  |  | TP2 vs TP4 | 0.895 | 4.607 | 0.071 | ***0.0002 | **0.0012 |
|  |  | TP3 vs TP4 | 0.252 | 1.299 | 0.021 | 0.0674 | 0.4044 |
|  | Weighted Unifrac | TP1 vs TP2 | 0.19 | 2.332 | 0.037 | **0.003 | *0.020 |
|  |  | TP1 vs TP3 | 0.222 | 2.62 | 0.042 | **0.001 | **0.0072 |
|  |  | TP1 vs TP4 | 0.22 | 2.467 | 0.039 | **0.002 | *0.012 |
|  |  | TP2 vs TP3 | 0.233 | 2.885 | 0.046 | **0.001 | **0.006 |
|  |  | TP2 vs TP4 | 0.269 | 3.145 | 0.05 | ***0.0002 | **0.001 |
|  |  | TP3 vs TP4 | 0.08 | 0.898 | 0.015 | 0.529 | 1 |
| Cluster 2 | Unweighted Unifrac | TP1 vs TP2 | 0.74 | 4.114 | 0.137 | ***0.0002 | **0.0012 |
|  |  | TP1 vs TP3 | 0.789 | 4.166 | 0.138 | ***0.0002 | **0.0012 |
|  |  | TP1 vs TP4 | 0.551 | 2.82 | 0.098 | ***0.0004 | **0.0024 |
|  |  | TP2 vs TP3 | 0.555 | 3.405 | 0.116 | ***0.0002 | **0.0012 |
|  |  | TP2 vs TP4 | 0.484 | 2.861 | 0.099 | ***0.0002 | **0.0012 |
|  |  | TP3 vs TP4 | 0.402 | 2.247 | 0.08 | ***0.0002 | **0.0012 |
|  | Weighted Unifrac | TP1 vs TP2 | 0.405 | 5.353 | 0.171 | ***0.0002 | **0.0012 |
|  |  | TP1 vs TP3 | 0.266 | 2.751 | 0.096 | **0.0014 | **0.0084 |
|  |  | TP1 vs TP4 | 0.268 | 3.187 | 0.109 | ***0.0006 | **0.0036 |
|  |  | TP2 vs TP3 | 0.257 | 2.962 | 0.102 | **0.0016 | **0.0096 |
|  |  | TP2 vs TP4 | 0.233 | 3.146 | 0.108 | **0.0026 | *0.0156 |
|  |  | TP3 vs TP4 | 0.249 | 2.618 | 0.091 | **0.003 | *0.018 |
| Cluster 3 | Unweighted Unifrac | TP1 vs TP2 | 0.245 | 1.319 | 0.022 | *0.023 | 0.138 |
|  |  | TP1 vs TP3 | 0.74 | 3.612 | 0.059 | ***0.0002 | **0.0012 |
|  |  | TP1 vs TP4 | 1.188 | 6.607 | 0.102 | ***0.0002 | **0.0012 |
|  |  | TP2 vs TP3 | 0.719 | 3.406 | 0.055 | ***0.0002 | **0.0012 |
|  |  | TP2 vs TP4 | 1.234 | 6.644 | 0.103 | ***0.0002 | **0.0012 |
|  |  | TP3 vs TP4 | 0.61 | 2.974 | 0.049 | ***0.0002 | **0.0012 |
|  | Weighted Unifrac | TP1 vs TP2 | 0.144 | 2.107 | 0.035 | *0.01 | 0.06 |
|  |  | TP1 vs TP3 | 0.317 | 3.851 | 0.062 | ***0.0002 | **0.0012 |
|  |  | TP1 vs TP4 | 0.344 | 4.919 | 0.078 | ***0.0002 | **0.0012 |
|  |  | TP2 vs TP3 | 0.292 | 3.428 | 0.056 | ***0.0002 | **0.0012 |
|  |  | TP2 vs TP4 | 0.371 | 5.105 | 0.081 | ***0.0002 | **0.0012 |
|  |  | TP3 vs TP4 | 0.295 | 3.401 | 0.055 | ***0.0004 | **0.0024 |

R^2^ and p values shown were calculated by Adonis test and 4999 permutations using the unweighted and weighted Unifrac distance. p values were adjusted by Bonferroni’s multiple pairwise tests and presented as *** p < 0.001, ** p ≥ 0.001 - < 0.01, * p ≥ 0.01- 0.05. TP= Timepoint.

Table S16: Results of Friedman rank-sum and post hoc Nemenyi multiple pairwise comparison tests of major faecal bacteria of Cluster 1 across the time points

| Bacteria | Friedman rank sum test p value | Nemenyi post hoc p-value | | | | | |
| --- | --- | --- | --- | --- | --- | --- | --- |
|  |  | Timepoint 1 vs Timepoint 2 | Timepoint 1 vs Timepoint 3 | Timepoint 1 vs Timepoint 4 | Timepoint 2 vs Timepoint 3 | Timepoint 2 vs Timepoint 4 | Timepoint 3 vs Timepoint 4 |
| *Lactobacillus* | ****2.24E-08 | ***0.001 | ****4.04E-06 | 0.979 | 0.700 | **0.003 | ****2.83E-05 |
| *Megasphaera* | **0.007 | 0.066 | 0.901 | *0.017 | 0.287 | 0.961 | 0.107 |
| *Lachnospiraceae* | *0.033 | 0.133 | 0.979 | 0.085 | 0.287 | 0.997 | 0.200 |
| *Enterobacteriaceae* | *0.049 | 0.576 | 0.514 | 0.901 | *0.040 | 0.935 | 0.164 |
| *Phascolarctobacterium* | 0.074 |  |  |  |  |  |  |
| *Faecalibacterium* | 0.113 |  |  |  |  |  |  |
| *Streptococcus* | 0.113 |  |  |  |  |  |  |
| *Oscillospira* | 0.233 |  |  |  |  |  |  |
| *Ruminococcaceae* | 0.257 |  |  |  |  |  |  |
| *Blautia* | 0.265 |  |  |  |  |  |  |
| *Bacteroides* | 0.320 |  |  |  |  |  |  |
| *Megamonas* | 0.379 |  |  |  |  |  |  |
| *[Eubacterium]* | 0.436 |  |  |  |  |  |  |
| *Sutterella* | 0.463 |  |  |  |  |  |  |
| Clostridiales | 0.505 |  |  |  |  |  |  |
| *Parabacteroides* | 0.566 |  |  |  |  |  |  |
| *Ruminococcus* | 0.582 |  |  |  |  |  |  |
| *Erysipelotrichaceae* | 0.582 |  |  |  |  |  |  |
| *Lachnospira* | 0.637 |  |  |  |  |  |  |
| *Collinsella* | 0.640 |  |  |  |  |  |  |
| *Bifidobacterium* | 0.662 |  |  |  |  |  |  |
| *[Ruminococcus]* | 0.667 |  |  |  |  |  |  |
| *Prevotella* | 0.691 |  |  |  |  |  |  |
| Clostridiales.Other.Other | 0.767 |  |  |  |  |  |  |
| *Coprococcus* | 0.888 |  |  |  |  |  |  |
| *Dorea* | 0.947 |  |  |  |  |  |  |

p values calculated from Friedman rank-sum and post hoc Nemenyi multiple pairwise comparison tests are described. p values of Nemenyi test were not stated if p values of Friedman test were > 0.05. The bacteria which were significantly different between each time point are presented as **** p < 0.0001, *** p ≥ 0.0001 - < 0.001, ** p ≥ 0.001 - < 0.01, * p ≥ 0.01- 0.05.

Table S17: Results of Friedman rank-sum and post hoc Nemenyi multiple pairwise comparison tests of major faecal bacteria of Cluster 2 across the time points

| Bacteria | Friedman rank sum test p value | Nemenyi post hoc p-value | | | | | |
| --- | --- | --- | --- | --- | --- | --- | --- |
|  |  | Timepoint 1 vs Timepoint 2 | Timepoint 1 vs Timepoint 3 | Timepoint 1 vs Timepoint 4 | Timepoint 2 vs Timepoint 3 | Timepoint 2 vs Timepoint 4 | Timepoint 3 vs Timepoint 4 |
| *Prevotella* | **0.008 | 0.991 | 0.884 | *0.011 | 0.972 | *0.028 | 0.089 |
| Clostridiales | **0.010 | 0.062 | 0.972 | 0.991 | *0.018 | *0.028 | 0.999 |
| *Lactobacillus* | *0.012 | 0.735 | 0.816 | 0.227 | 0.999 | *0.018 | *0.028 |
| *Collinsella* | *0.020 | 0.295 | 0.936 | 0.645 | 0.089 | *0.018 | 0.936 |
| *Erysipelotrichaceae* | *0.033 | 0.295 | 1.000 | 0.089 | 0.295 | 0.936 | 0.089 |
| *Megasphaera* | *0.034 | 0.552 | 0.991 | 0.373 | 0.373 | *0.018 | 0.552 |
| *[Ruminococcus]* | 0.059 |  |  |  |  |  |  |
| *Lachnospira* | 0.093 |  |  |  |  |  |  |
| *Lachnospiraceae* | 0.116 |  |  |  |  |  |  |
| *Bifidobacterium* | 0.125 |  |  |  |  |  |  |
| *Faecalibacterium* | 0.180 |  |  |  |  |  |  |
| Clostridiales.Other.Other | 0.224 |  |  |  |  |  |  |
| *Blautia* | 0.258 |  |  |  |  |  |  |
| *Enterobacteriaceae* | 0.297 |  |  |  |  |  |  |
| *Megamonas* | 0.392 |  |  |  |  |  |  |
| *Sutterella* | 0.405 |  |  |  |  |  |  |
| *Ruminococcus* | 0.543 |  |  |  |  |  |  |
| *Oscillospira* | 0.672 |  |  |  |  |  |  |
| *Coprococcus* | 0.733 |  |  |  |  |  |  |
| *Phascolarctobacterium* | 0.737 |  |  |  |  |  |  |
| *[Eubacterium]* | 0.766 |  |  |  |  |  |  |
| *Dorea* | 0.877 |  |  |  |  |  |  |
| *Bacteroides* | 0.896 |  |  |  |  |  |  |
| *Streptococcus* | 0.896 |  |  |  |  |  |  |
| *Parabacteroides* | 0.968 |  |  |  |  |  |  |
| *Ruminococcaceae* | 0.319 |  |  |  |  |  |  |

p values calculated from Friedman rank-sum and post hoc Nemenyi multiple pairwise comparison tests are described. p values of Nemenyi test were not stated if p values of Friedman test were > 0.05. The bacteria which were significantly different between each time point are presented as **** p < 0.0001, *** p ≥ 0.0001 - < 0.001, ** p ≥ 0.001 - < 0.01, * p ≥ 0.01- 0.05.

Table S18: Results of Friedman rank-sum and post-hoc Nemenyi multiple pairwise comparison tests of major faecal bacteria of Cluster 3 across the time points

| Bacteria | Friedman rank sum test p value | Nemenyi post-hoc comparison test | | | | | |
| --- | --- | --- | --- | --- | --- | --- | --- |
|  |  | Timepoint 1 vs Timepoint 2 | Timepoint 1 vs Timepoint 3 | Timepoint 1 vs Timepoint 4 | Timepoint 2 vs Timepoint 3 | Timepoint 2 vs Timepoint 4 | Timepoint 3 vs Timepoint 4 |
| *Lactobacillus* | ****2.69E-09 | **0.001 | ***0.0001 | 0.690 | 0.959 | ****9.44E-06 | ****6.93E-07 |
| *Bifidobacterium* | ****1.67E-05 | 0.932 | 0.991 | ***0.0002 | 0.805 | **0.003 | ****6.39E-05 |
| *Collinsella* | ***0.0001 | 0.991 | 1.000 | **0.001 | 0.991 | **0.004 | **0.001 |
| *Phascolarctobacterium* | **0.003 | *0.046 | 0.437 | **0.003 | 0.690 | 0.805 | 0.188 |
| *Sutterella* | 0.059 |  |  |  |  |  |  |
| *Enterobacteriaceae* | 0.081 |  |  |  |  |  |  |
| *Parabacteroides* | 0.083 |  |  |  |  |  |  |
| *Bacteroides* | 0.104 |  |  |  |  |  |  |
| *Lachnospiraceae* | 0.181 |  |  |  |  |  |  |
| *Faecalibacterium* | 0.184 |  |  |  |  |  |  |
| *[Eubacterium]* | 0.184 |  |  |  |  |  |  |
| *Blautia* | 0.204 |  |  |  |  |  |  |
| Clostridiales | 0.245 |  |  |  |  |  |  |
| *Ruminococcus* | 0.249 |  |  |  |  |  |  |
| *Prevotella* | 0.267 |  |  |  |  |  |  |
| *Oscillospira* | 0.392 |  |  |  |  |  |  |
| Clostridiales.Other.Other | 0.404 |  |  |  |  |  |  |
| *Streptococcus* | 0.715 |  |  |  |  |  |  |
| *Erysipelotrichaceae* | 0.760 |  |  |  |  |  |  |
| *Megasphaera* | 0.821 |  |  |  |  |  |  |
| *Ruminococcaceae* | 0.821 |  |  |  |  |  |  |
| *Megamonas* | 0.853 |  |  |  |  |  |  |
| *Coprococcus* | 0.878 |  |  |  |  |  |  |
| *[Ruminococcus]* | 0.914 |  |  |  |  |  |  |
| *Dorea* | 0.940 |  |  |  |  |  |  |
| *Lachnospira* | 0.940 |  |  |  |  |  |  |

p values calculated from Friedman rank-sum and post hoc Nemenyi multiple pairwise comparison tests are described. p values of Nemenyi test were not stated if p values of Friedman test were > 0.05. The bacteria which were significantly different between each time point are presented as **** p < 0.0001, *** p ≥ 0.0001 - < 0.001, ** p ≥ 0.001 - < 0.01, * p ≥ 0.01- 0.05.

Table S19: Average concentration of faecal water cytokines (pg/g of wet weight of faeces) in all clusters throughout the time points (a) Timepoint 1 (b) Cluster 1 (c) Cluster 2 (d) Cluster 3

| Cytokines | (a) Timepoint 1 Mean ± SD (pg/g of wet weight of faeces) | | | | |
| --- | --- | --- | --- | --- | --- |
|  | Cluster 1 | Cluster 2 | Cluster 3 | Cluster 2/Cluster1 | Cluster 3/Cluster 1 |
| IL-2 | 272.48 ± 532.25 | 987.23 ± 678.55 | 506.81 ± 724.33 | 3.62 | 0.51 |
| IL-1β | 55.63 ± 173.79 | 88.71 ± 225.12 | 558.38 ± 1598.87 | 1.59 | 6.29 |
| IL-8 | 235.61 ± 721.5 | 289.01 ± 498.38 | 475.25 ± 1405.2 | 1.23 | 1.64 |
| TNFα | 113.45 ± 355.39 | 372.15 ± 651.99 | 355.54 ± 670.01 | 3.28 | 0.96 |
| IL-12 | 37.03 ± 144.47 | 229.25 ± 348.43 | 173.56 ± 336.99 | 6.19 | 0.76 |
| IL-5 | 0 ± 0 | 0 ± 0 | 7.36 ± 27.05 | ND | ND |
| IFNɣ | 7.11 ± 28.91 | 0 ± 0 | 18.33 ± 47.32 | 0 | ND |
| IL-4 | 3.4 ± 18.62 | 0 ± 0 | 25.09 ± 44.41 | 0 | ND |
| IL-10 | 1.58 ± 8.65 | 0 ± 0 | 15.73 ± 24.83 | 0 | ND |
| GM-CSF | 4.71 ± 11.89 | 0 ± 0 | 6.2 ± 16.81 | 0 | ND |
| IL-6 | 0 ± 0 | 0 ± 0 | 0.92 ± 4.89 | ND | ND |

ND= Not Determined. IL= Interleukin, TNF= Tumour necrosis factor, IFN= Interferon, GM-CSF= Granulocyte-macrophage-stimulating factor, SD= standard deviation.

| (b) Cluster 1 Mean ± SD (pg/g of wet weight of faeces) | | | | |
| --- | --- | --- | --- | --- |
| Cytokines | Timepoint 1 | Timepoint 2 | Timepoint 3 | Timepoint 4 |
| IL-2 | 272.48 ± 532.25 | 304.37 ± 532.5 | 321.47 ± 580.95 | 316.82 ± 524.5 |
| IL-1β | 55.63 ± 173.79 | 681.93 ± 3288.75 | 68.16 ± 119.66 | 233.32 ± 743.26 |
| IL-8 | 235.61 ± 721.5 | 190.78 ± 308.78 | 216.02 ± 298.02 | 239.07 ± 403.48 |
| TNFα | 113.45 ± 355.39 | 97.6 ± 319.96 | 132.42 ± 374.41 | 165.15 ± 581.52 |
| IL-12 | 37.03 ± 144.47 | 15.91 ± 78.07 | 116 ± 247.81 | 66.83 ± 209.36 |
| IL-5 | 0.00 ± 0.00 | 0.00 ± 0.00 | 8.61 ± 24.41 | 9.53 ± 20.32 |
| IFNɣ | 7.11 ± 28.91 | 8.93 ± 31.83 | 9.08 ± 28.08 | 31.52 ± 85.1 |
| IL-4 | 3.4 ± 18.62 | 3.95 ± 18.83 | 3.55 ± 15.04 | 6.42 ± 32.78 |
| IL-10 | 1.58 ± 8.65 | 2.27 ± 12.45 | 0.82 ± 4.46 | 5.43 ± 23.37 |
| GM-CSF | 4.71 ± 11.89 | 0.79 ± 4.34 | 0.00 ± 0.00 | 8.86 ± 32.26 |
| IL-6 | 0.00 ± 0.00 | 0.35 ± 1.93 | 0.00 ± 0.00 | 5.02 ± 27.5 |

| (c) Cluster 2 Mean ± SD (pg/g of wet weight of faeces) | | | | |
| --- | --- | --- | --- | --- |
| Cytokines | Timepoint 1 | Timepoint 2 | Timepoint 3 | Timepoint 4 |
| IL-2 | 987.23 ± 678.55 | 866.96 ± 808.32 | 570.43 ± 695.64 | 722.13 ± 788.73 |
| IL-1β | 88.71 ± 225.12 | 95.4 ± 237.4 | 800.67 ± 2991.99 | 641.03 ± 2099.72 |
| IL-8 | 289.01 ± 498.38 | 308.73 ± 368.57 | 360.52 ± 1192.21 | 76.74 ± 138.2 |
| TNFα | 372.15 ± 651.99 | 522.31 ± 776.01 | 180.78 ± 459.91 | 503.48 ± 772.05 |
| IL-12 | 229.25 ± 348.43 | 201.42 ± 404.27 | 108.84 ± 217.94 | 212.92 ± 359.75 |
| IL-5 | 0.00 ± 0.00 | 81.08 ± 303.39 | 0.00 ± 0.00 | 0.00 ± 0.00 |
| IFNɣ | 0.00 ± 0.00 | 3.92 ± 14.67 | 0.00 ± 0.00 | 0.00 ± 0.00 |
| IL-4 | 0.00 ± 0.00 | 10.93 ± 38.33 | 0.00 ± 0.00 | 7.71 ± 28.86 |
| IL-10 | 0.00 ± 0.00 | 43.41 ± 162.41 | 0.00 ± 0.00 | 4.47 ± 16.73 |
| GM-CSF | 0.00 ± 0.00 | 0.00 ± 0.00 | 0.00 ± 0.00 | 3.07 ± 11.49 |
| IL-6 | 0.00 ± 0.00 | 0.00 ± 0.00 | 0.00 ± 0.00 | 0.00 ± 0.00 |

| (d) Cluster 3 Mean ± SD (pg/g of wet weight of faeces) | | | | |
| --- | --- | --- | --- | --- |
| Cytokines | Timepoint 1 | Timepoint 2 | Timepoint 3 | Timepoint 4 |
| IL-2 | 506.81 ± 724.33 | 446.55 ± 641.26 | 483.66 ± 672.13 | 520.52 ± 760.58 |
| IL-1β | 558.38 ± 1598.87 | 146.49 ± 291.52 | 1034.57 ± 3263.82 | 148.44 ± 310.56 |
| IL-8 | 475.25 ± 1405.2 | 284.84 ± 528.72 | 237.48 ± 568.56 | 219.51 ± 446.5 |
| TNFα | 355.54 ± 670.01 | 171.97 ± 445.47 | 278 ± 498.76 | 367.3 ± 676.38 |
| IL-12 | 173.56 ± 336.99 | 99.04 ± 251.61 | 181.93 ± 284.1 | 180.95 ± 380.17 |
| IL-5 | 7.36 ± 27.05 | 0.00 ± 0.00 | 46.8 ± 241.06 | 88.75 ± 303.61 |
| IFNɣ | 18.33 ± 47.32 | 13.46 ± 52.28 | 32.41 ± 83.92 | 3.45 ± 18.26 |
| IL-4 | 25.09 ± 44.41 | 16.23 ± 35.43 | 31.53 ± 64.71 | 25.46 ± 49.27 |
| IL-10 | 15.73 ± 24.83 | 8.33 ± 24.06 | 17.48 ± 35.36 | 9.56 ± 24.81 |
| GM-CSF | 6.2 ± 16.81 | 6.7 ± 17.77 | 15.01 ± 30.27 | 7.66 ± 22.6 |
| IL-6 | 0.92 ± 4.89 | 2.16 ± 11.24 | 4.11 ± 15.11 | 0.00 ± 0.00 |

**Supplementary Text S1**

Food Frequency Questionnaire

Project ID no:

Age:

Race: Chinese / Malay / Indian / Eurasian / Others:

Gender: M / F

Height (in m):

Body weight (in kg):

Body Mass Index (BMI):

*BMI = Mass(in kg)/ Height x Height (in m)*

Please tick the most appropriate answer

| **Section A (Personal information)** | | **Yes** | **No** | **If yes, please specify** |
| --- | --- | --- | --- | --- |
| **1.** | **Are you currently taking any medication within the past 7 days?** |  |  |  |
| **2.** | **Has your diet changed over the past year? (EG: became a Vegetarian)** |  |  |  |
| **3.** | **Do you have any dietary requirements? (EG: Vegetarian, Halal, Kosher, etc)** |  |  |  |

| **Section B (Consumption of fermented food products)** | | **Yes** | **No** | **If yes, how often?** |
| --- | --- | --- | --- | --- |
| **1.** | **Do you consume fermented vegetables? (EG: Kimchi, pickles, etc)** |  |  | **time(s) per week** |
| **2.** | **Do you consume fermented dairy products? (EG: Yogurt, cheese, fermented milk, etc)** |  |  | **time(s) per week** |
| **3.** | **Do you consume fermented beverages? (EG: Wine, stout beer, sakae, yakult, etc)** |  |  | **bottle(s)/cup(s) per week** |
| **4.** | **Do you consume fermented condiments?**  **(EG: Soya sauce, sour sauce, tabasco sauce, vinegar, ketchup)** |  |  | **time(s) per day** |
| **5.** | **Do you consume fermented snacks?**  **(EG: Chocolate, probiotic ice cream, probiotic cheesecake, nata de coco etc)** |  |  | **packet(s) per week** |
| **6.** | **Do you consume other Types of fermented food that does not fit into the categories in questions 1 - 5? (EG: Cod Liver Oil (Traditional one), fermented tofu, Katuobushi (the topping on takoyaki))** |  |  | **time(s) per week** |
| **7.** | **Others, please specify:** |  |  |  |

| **Section C (Consumption of carbohydrates, proteins and fats)** | | **Please list them down in this column** | |
| --- | --- | --- | --- |
| **1.** | **Where is your main source of carbohydrate?**  **(EG: Wheat-based bread, cereal, rice, pastries, noodles, etc)** |  | **time(s) per week**  **time(s) per week**  **time(s) per week**  **time(s) per week** |
| **2.** | **Where is your main source of protein?**  **(EG: Chicken, duck, beef, pork, mutton, seafood, soy-based products, legumes, eggs, etc)** |  | **time(s) per week**  **time(s) per week**  **time(s) per week**  **time(s) per week** |
| **3.** | **Where is your main source of fat?**  **(EG: Nuts, seeds, fish, plant-based oils, dairy products, etc)** |  | **time(s) per week**  **time(s) per week**  **time(s) per week**  **time(s) per week** |
| **4** | **What Type of oil do you usually consume at home? (EG: olive oil, canola oil, sunflower seed oil, etc)** |  | **tsp(s) per day** |
| **4.** | **Do you consume supplements? (EG: Vitamin tablets,** |  | **pill(s)/tablets/** |
|  | **protein shake)** |  | **cups per day** |
|  | **If yes, please state the supplements which you are** |  |  |
|  | **taking.** |  |  |

| **Section D (Consumption of vegetables, fruits, nuts)** | | **Please list them down in this column** | |
| --- | --- | --- | --- |
| **1.** | **Vegetables** |  | **time(s) per week**  **time(s) per week**  **time(s) per week**  **time(s) per week** |
| **2.** | **Fruits** |  | **time(s) per week**  **time(s) per week**  **time(s) per week**  **time(s) per week** |
| **3.** | **Nuts** |  | **time(s) per week**  **time(s) per week**  **time(s) per week**  **time(s) per week** |

| **Section E (Consumption of fats)** | | **Yes** | **No** | **If yes, how often?** |
| --- | --- | --- | --- | --- |
| **1.** | **Do you eat deep fried food?** |  |  | **time(s) per week** |
| **2.** | **Do you eat pan fry food?** |  |  | **time(s) per week** |
| **3.** | **Do you eat steamed food?** |  |  | **time(s) per week** |
| **4.** | **Do you eat meat with skin?** |  |  | **time(s) per week** |
| **5.** | **Do you often opt for fatty parts of meat?** |  |  | **time(s) per week (Include examples)** |
| **5.** | **Others, please specify:** |  |  |  |

| **Section F (Consumption of beverages)** | | **Yes** | **No** | **If yes, how often?** |
| --- | --- | --- | --- | --- |
| **1.** | **Do you drink tea?** |  |  | **cup(s) per day** |
| **2.** | **Do you drink coffee?** |  |  | **cup(s) per day** |
| **3.** | **Do you drink soft drinks?**  **(EG: Ribena, ice lemon tea, coke)** |  |  | **bottle(s)/can(s) per week** |
| **4.** | **Do you drink alcohol?**  **(EG: Vodka, whiskey, wine, champagne, beer)** |  |  | **glass(es) per week** |
| **5.** | **Do you drink fruit juices?** |  |  | **cup(s) per week** |
| **6.** | **Do you drink any dairy products? (EG: Milk)** |  |  | **cup(s) per week** |
| **7.** | **Others, please specify:** |  |  |  |

| **Section G (Consumption of fast food)** | | **Yes** | **No** | **If yes, how often?** |
| --- | --- | --- | --- | --- |
| **1.** | **Do you consume fast food? (EG: French fries, burger, pizza)** |  |  | **time(s) per week** |

| **Section H (Consumption of spice, herbs and condiments)** | | **Yes** | **No** | **If yes, how often?** |
| --- | --- | --- | --- | --- |
| **1.** | **Do you consume spices?**  **(EG: Chilli, nutmeg, cinnamon)** |  |  | **time(s) per day** |
| **2.** | **Do you consume herbs? (EG: Ginseng, basil, oregano)** |  |  | **time(s) per week** |
| **3.** | **Do you consume condiments?**  **(EG: Ketchup, mustard, BBQ sauce, sour cream, thousand island, mayonnaise)** |  |  | **time(s) per day** |

**Annex**

| **Carbohydrates** |
| --- |
| Brown rice |
| Purple rice |
| Glutinous rice |
| Yellow noodles |
| White noodles |
| Buckwheat (soba) noodles |
| Cellophane noodles |
| Green tea noodles |
| Udon |
| Egg-based noodles (Ramen, banmian) |
| E mee |
| Wholemeal bread |
| Wheat-based bread |
| Multigrain bread |
